# Clathrin Heavy Chain-Linked Opioid Receptor Regulation in Burn Patients

**DOI:** 10.1093/jbcr/irag033.576

**Published:** 2026-04-06

**Authors:** Kristin N Grimsrud, Tajia L Green, David Wang, Aijun Wang, Tina L Palmieri

**Affiliations:** University of California Davis, Sacramento, CA; Shriners Children's Northern California, Sacramento, CA; University of California Davis, Sacramento, CA; University of California Davis School of Medicine, Sacramento, CA; University of California Davis Health, Shriners Children’s - Northern California, Sacramento, CA

## Abstract

**Background/Objective:**

Severe burn injury is associated with profound inflammatory, metabolic, and neuroimmune responses that contribute to persistent pain and reduced opioid responsiveness. Although dysregulation of μ-opioid receptor (MOR) signaling and opioid tolerance development are well-known in burn patients, the systemic molecular processes underlying these changes are unclear. Extracellular vesicles (EVs) carry protein cargo that reflects cellular signaling and trafficking pathways and provide a systemic window into receptor regulatory mechanisms. We hypothesized that EV proteomic profiling would identify changes in proteins mapping to opioid signaling pathways that reflect altered opioid receptor function regulation following burn injury.

**Methods:**

Plasma EVs from 37 burn patients with >15% total body surface area burn and 21 nonburn controls were analyzed by quantitative proteomics. Differential protein abundance was integrated into pathway-level analysis using Ingenuity Pathway Analysis (IPA), with expression changes represented as log2 fold change (Expr Log Ratio). Proteins mapping to the opioid signaling pathway were examined to characterize patterns in receptor signaling and trafficking.

**Results:**

Multiple proteins mapping to the opioid signaling pathway demonstrated coordinated differences in EV cargo. Notably, clathrin heavy chain (CLTC), a central component of clathrin-mediated endocytosis, was robustly enriched (~3-fold), while the adaptor protein AP2A2 demonstrated reduced representation. Small GTPases involved in membrane dynamics and vesicle trafficking, including RAC1, RALB, and RAP1B, demonstrated modest increases. Canonical G protein signaling components and calcium channel–associated proteins exhibited minimal directional changes. This pattern suggests that changes within opioid pathway–associated proteins are dominated by molecules involved in receptor internalization, membrane trafficking, and vesicle dynamics rather than uniform modulation of classical GPCR second-messenger signaling.

**Conclusions:**

Clathrin-dependent trafficking and adaptor-mediated internalization are established regulators of MOR desensitization, recycling, and tolerance. Our EV proteomic results suggest prominent remodeling of receptor regulatory and trafficking pathways. Given that patients received opioids throughout hospitalization, these findings may reflect the combined effects of burn-induced systemic responses and opioid-driven adaptations in receptor regulatory mechanisms.

**Applicability of Research to Practice:**

Together, these data provide new insight into molecular pathways linked to altered opioid responsiveness following burn injury and highlight receptor trafficking pathways as potential targets for improving analgesic strategies and reduction of tolerance.

**Funding for the study:**

Academic and Department Seed Grants.

